# Stalls in Africa’s fertility decline partly result from disruptions in female education

**DOI:** 10.1073/pnas.1717288116

**Published:** 2019-02-04

**Authors:** Endale Kebede, Anne Goujon, Wolfgang Lutz

**Affiliations:** ^a^Wittgenstein Centre for Demography and Global Human Capital (IIASA, VID/OeAW, WU), International Institute for Applied Systems Analysis, 2361 Laxenburg, Austria

**Keywords:** fertility, sub-Saharan Africa, educational discontinuity, macro-economic crisis, population projections

## Abstract

Population projections for sub-Saharan Africa have, over the past decade, been corrected upwards because in a number of countries, the earlier declining trends in fertility stalled around 2000. While most studies so far have focused on economic, political, or other factors around 2000, here we suggest that in addition to those period effects, the phenomenon also matched up with disruptions in the cohort trends of educational attainment of women after the postindependence economic and political turmoil. Disruptions likely resulted in a higher proportion of poorly educated women of childbearing age in the late 1990s and early 2000s than there would have been otherwise. In addition to the direct effects of education on lowering fertility, these less-educated female cohorts were also more vulnerable to adverse period effects around 2000. To explore this hypothesis, we combine individual-level data from Demographic and Health Surveys for 18 African countries with and without fertility stalls, thus creating a pooled dataset of more than two million births to some 670,000 women born from 1950 to 1995 by level of education. Statistical analyses indicate clear discontinuities in the improvement of educational attainment of subsequent cohorts of women and stronger sensitivity of less-educated women to period effects. We assess the magnitude of the effect of educational discontinuity through a comparison of the actual trends with counterfactual trends based on the assumption of no education stalls, resulting in up to half a child per woman less in 2010 and 13 million fewer live births over the 1995–2010 period.

## Significance

The future pace of fertility decline in sub-Saharan Africa is the main determinant of future world population growth and will have massive implications for Africa and the rest of the world, not least through international migration pressure and difficulties in meeting the sustainable development goals. In this context, there have been concerns about recent stalls in the fertility decline in some African countries. Our findings suggest that these stalls are in part explained by earlier stalls in female education and that less-educated women are more vulnerable to adverse period conditions. This has important implications for setting policy priorities.

All human populations have entered the process of demographic transition in which first, death rates start to fall due to socioeconomic development and improved public health, and then after some time lag, birth rates start to decline. During the period when death rates are already low and birth rates are still high, populations grow rapidly. This was the case in Europe and North America around 1900, and the process subsequently spread to Latin America and eastern Asia, and then to southern and western Asia. In most of these regions, fertility rates have already fallen to low levels, even when the population still continues to grow due to the age-structural momentum in which larger cohorts of young women still enter the reproductive ages and death rates continue to fall.

**Fig. 1.**
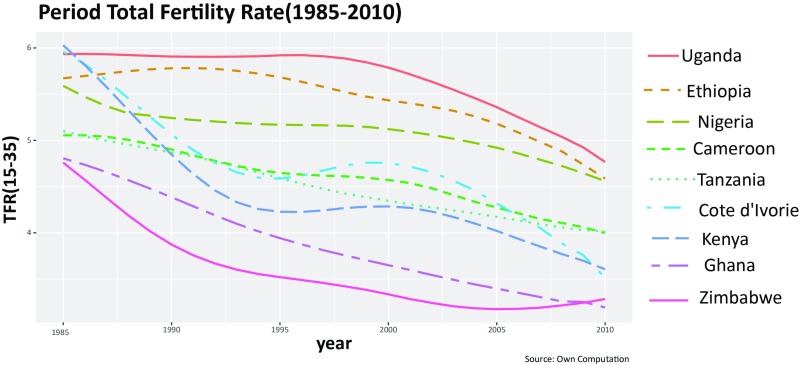
Reconstructed period TFRs (for women aged 15 to 35 y) for nine selected countries in sub-Saharan Africa for 1985–2010 based on successive DHSs.

Sub-Saharan Africa has been the last world region to enter this demographic transition. It was only in the 1980s that birth rates started to fall in most countries, but these declines have been uneven and have stalled at times. Particularly in the late 1990s and early 2000s, some sub-Saharan African countries have experienced a leveling off of their fertility decline and, in some cases, even saw a reversal, leading to an increase (as shown in Fig. 1 for selected countries included in our dataset). Much has been written and speculated about this so-called stalled African fertility transition (1). The reasons for this interruption of the fertility decline in many sub-Saharan African countries have remained a demographic mystery because little consensus exists on the causes of the stalls (2). Most of the existing studies try to link the fertility stalls to some specific period factors such as the slower trends in socioeconomic development prevalent in the stalling countries (3), the low priority assigned to family planning programs at the beginning of the 21st century (4, 5), the impact of HIV/AIDS mainly through its effect on child mortality (2, 6), and other factors related to public and reproductive health. In contrast to these explanations, recently, Goujon et al. (7) proposed another plausible explanation, focusing on cohort effects rather than period effects. These authors linked the fertility stalls around 2000 to the fact that some cohorts of women were subject to an education stall, possibly associated with the adverse effects on education of the structural adjustment programs (SAPs) launched by the Bretton Woods Institutions in the 1980s.

The analysis presented in this paper provides a more comprehensive assessment on the basis of microlevel data from 18 African countries and highlights the cohort and period effects that, in combination, could have resulted in the slow-down in the decline of period fertility around 2000 in some countries. The discontinuity in cohort trends of improving educational attainment (due to the economic and political turmoil in some countries around the 1980s) is consistent with the higher proportion of poorly educated women of childbearing age in the late 1990s and early 2000s than there would have been without these education disruptions. This phenomenon coupled with the relatively higher vulnerability of less-educated women to period effects has likely contributed to the stalls in the period fertility declines.

## Education and Fertility Discontinuities in the Context of Crises in Africa

The postindependence period was a time of great expectations in sub-Saharan Africa, as most countries engaged in a process of expansion of social services (8). However, the initial period of economic and social bliss was soon replaced by harsher times related to the consequence of external shocks of oil price increases, declining terms of trade, and increases in interest rates that had a massive impact on most African economies, increasing government external debt (9). This resulted in austerity measures and massive cuts in government budgets, particularly in the social sectors of health and education, mostly in the framework of SAPs introduced in Africa by the International Monetary Fund (IMF) and the World Bank (9). These austerity measures affecting the social sector have been happening recurrently, not always with the same strength, throughout the last decades in most African countries. The particularity of the adjustment programs of the early 1980s was that they were mostly not accompanied by compensating programs such as safety net programs for the most vulnerable population—for example, cash transfers or school-based food programs—as was the case thereafter (mostly from the 1990s onward). It is impossible to disentangle whether the SAP or the initial dire situation (e.g., stressed government budgets and diminishing employment opportunities) is at the origin of the slow-down in educational improvement that we observe in our cohort approach. Nevertheless, it is clear that the unfortunate women who, in their childhood in the 1980s, were deprived of their education opportunities will bear the consequences of this over their entire life cycle. These life-long consequences not only concern health and income, they could also be relevant for fertility, which in time would affect population growth and society at large.

In the literature so far, fertility trends in sub-Saharan Africa have hardly been linked to the slow-down in education progress. The two phenomena are, indeed, almost two decades apart, which may have seemed too long for any direct causal effect from, for example, reduced reproductive health spending on fertility. This 20-y lag is, however, precisely the timing that would be expected for an effect on fertility operating through female education: declining primary school enrolment rates for girls during the 1980s would result in lower education, and hence higher fertility, for women in their prime childbearing ages around 2000. Given the strong differentials in fertility by level of female education in all African countries and the extensive body of literature that explains the causal mechanisms behind the pervasive negative association between the two (10–14), it seems to be a plausible hypothesis to assume a direct effect from the stalled trend in female education to the subsequent stall in fertility decline in the countries affected by the former.

In addition to this hypothesis or in combination with it (as we will show later), the fertility stalls in the early 2000s could also be linked to some period effects affecting the women of childbearing age, as mentioned in the *Introduction*. Mixed results are coming out of studies trying to assess the impact of various types of crises on fertility, especially in the African context in which increasing economic hardship may not necessarily lead to lower fertility (as it does in countries further advanced in demographic transition), because there are still strongly pronatalist norms and children are typically seen as a way to diversify risk. Also, increased fertility enhances the probability of having a certain number of surviving children in times of mortality crisis. But there is also evidence for opposite effects of crises on lowering fertility in certain cases, such as short-term economic crisis, extreme weather events, or epidemic diseases (15). Different patterns are also found in urban areas where household living costs associated with additional children are substantially higher than in rural areas.

The purpose of this study is to systematically assess the pattern of period and cohort changes in fertility and educational attainment, as well as other possible drivers of fertility, using the broadest available datasets. We investigate the link between longitudinal cohort education trends and longitudinal cohort specific fertility trends from consecutive Demographic and Health Surveys (DHSs) conducted in the region. We also apply multivariate statistical analyses, adding national-level indicators to microlevel data, to explore alternative explanations and explore the relative impact of the different potential determinants of fertility.

## Data Used and Cohorts Reconstructed

This study is primarily based on a pooled microdataset from a total of 72 DHSs collected in 18 sub-Saharan African countries over the years 1990–2016. Within each country, the surveys made use of a two-stage cluster sampling technique to collect comparable, reliable, and nationally representative data on living conditions and demographic characteristics of households. The 18 sub-Saharan African countries represent about 66% of the population of the region in 2015 (16). The selected countries are Benin, Burkina Faso, Côte d’Ivoire, Cameroon, Republic of the Congo, Democratic Republic of the Congo, Ethiopia, Gabon, Ghana, Guinea, Kenya, Niger, Nigeria, Malawi, Tanzania, Uganda, Zambia, and Zimbabwe. The DHSs collect and report full birth histories of women aged 15 to 49 y. The pooled dataset for the 18 countries under study includes 2,040,664 births to 670,449 women. For each of the countries studied, we used multiple surveys taken at different points in time, ranging from two to five surveys per country, with an average of around four.

DHS individual and household files were used to construct fertility histories by age and educational attainment of the mother by single-year birth cohorts. Since DHSs are sample surveys, the information gathered at different points in time for the same national-level cohorts of women is not necessarily identical. Hence, considerable effort was invested in reconstructing consistent series of age- and cohort-specific data for the period 1985–2010 covering the cohorts of women born between 1950 and 1995. Because of these limited time windows of data availability, we will only consider fertility rates between the ages of 15 and 35 y, which do cover most of the reproduction time over the life cycle. In the case that different surveys provided different information on fertility for the same cohorts, a weighted average of the different surveys was used (more details about the reconstruction procedures are included in *SI Appendix*).

## Intercohort Changes in Education and Fertility

The reconstructed period fertility trends given in Fig. 1 show interesting differences in fertility levels and patterns of change. While Uganda shows stable fertility at a very high level until the onset of the fertility transition around 2000, Ghana shows a smooth and uninterrupted fertility decline over the entire period of 1985–2010. Côte d’Ivoire, on the other hand, shows a clear reversal of the fertility decline, with increases from 1995 to 2000, followed by a continuation of the decline. The pattern in Kenya is similar but somewhat less pronounced. Other countries such as Nigeria and Cameroon show a flattening of the curve in the middle of the period but no real increase. Both Nigeria and Cameroon resumed the declining trend over the last decade.

**Fig. 2.**
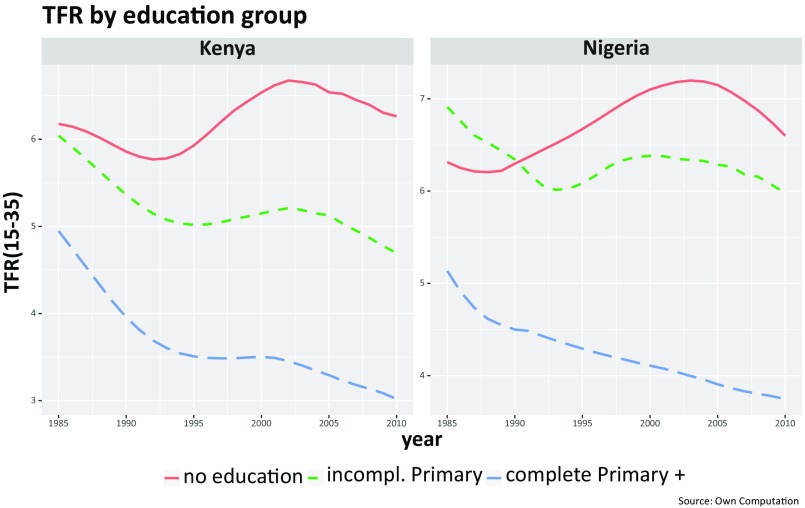
Reconstructed period TFRs (for women aged 15 to 35 y) for Kenya and Nigeria by level of education (no formal education vs. some formal education).

For two big countries with stalls in their fertility declines, Fig. 2 shows the reconstructed period fertility trends (1985–2010) by education group. The trends for all countries are given in *SI Appendix*, Fig. S3. We have categorized the educational attainment of women into three broad groups: no education, some primary education, and completed primary education or more. These two figures exhibit the well-documented education differences in fertility levels, with more-educated women wanting and actually having fewer children by using contraception more effectively compared with less-educated women (10). More interestingly, the stall in the period fertility decline and actual increases in some countries are more pronounced among the least educated groups. It would seem that uneducated women were more affected by some adverse period conditions prevailing in the late 1990s and early 2000s than better educated women.

**Fig. 3.**
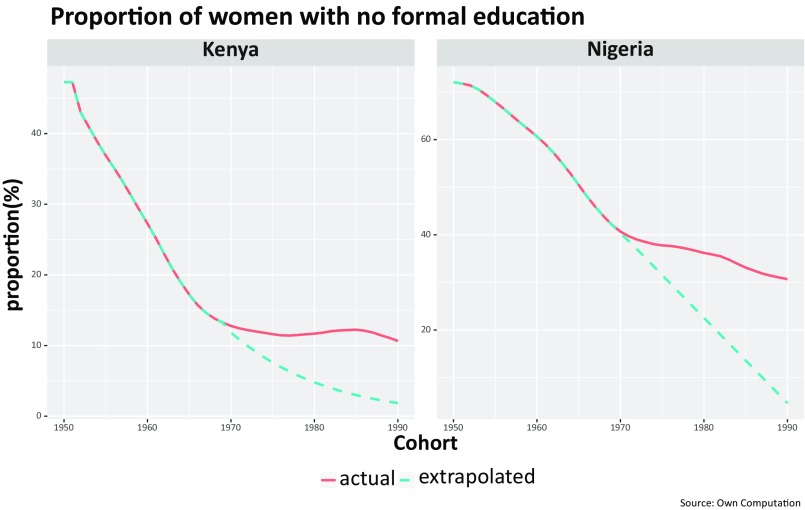
Reconstructed proportions of women with no formal education by cohorts born between 1950 and 1990 for Kenya and Nigeria (red line) and extrapolated trends for cohorts born after 1970 based on a hypothetical continuation of the trend of the earlier period (blue line).

There are thus two different mechanisms in which education could have influenced fertility in terms of cohort and period effects. The cohorts of women who had suffered a stall in their education progress in the context of postindependence economic and political problems had higher fertility than would have been the case without such stalls, due to the direct effect of education lowering fertility. However, in addition, the above-described impact of period effects on uneducated women around 2000 was also more sizable in terms of affecting more women because of a higher proportion of uneducated women. To have a deeper understanding of these patterns, we have taken a closer look at two examples: Nigeria as the most populous African country and Kenya as a country classified as “fertility stalled” by the large majority of previous studies and criteria. Fig. 2 shows the period fertility trends of those two countries by level of mothers’ education. In Kenya around 1995, women with completed primary or higher education had, on average, 3.5 children, whereas women without any schooling had 5.7 (a difference of 2.2 children). By 2005, the gap had significantly widened to 3.3 vs. 6.6 children (a difference of 3.3 children or exactly double that of women with at least completed primary education). Fig. 3 plots the trends in the proportions of women who never attended school by cohorts born between 1950 and 1990 and shows that in both countries, a clearly declining trend from birth cohort of 1950 to that of the 1970s was discontinued for the subsequent cohorts. For those born after 1970, something dramatic happened. The improving trend slowed markedly in Nigeria and even reversed in Kenya. Such education discontinuities were also observed in most other countries classified as fertility stalled, as shown in *SI Appendix*, Fig. S3.

This combination of a stall or even reversal in the cohort educational attainment and the above-described pronounced differences in fertility by education suggests a link between education and fertility stalls. It is not trivial, however, to estimate the size of this education-related cohort effect in relation to some also-evident period effects around the time of the stall. We do not choose a simple decomposition analysis because we should capture the joint implication of two independent forces: (*i*) the fact that without the education stall, more women would be in the educated category and, since these women would have lower fertility levels, overall fertility would be lower; and (*ii*) the fact that—as shown in Fig. 2—educated women are less affected by those period influences than women without any education. An appropriate way to assess the combined effect of these two different forces is to compare the actually observed fertility trends with a hypothetical or “counterfactual” trend based on the assumption of the absence of an education stall.

The blue dotted lines in Fig. 3 show this counterfactual trend in the proportions of women with no formal education, which was extrapolated from an autoregressive moving average model, based on the empirical trends of cohort educational attainment up to 1970. In other words, this counterfactual line indicates the improvement in education by cohorts for the hypothetical case that education levels would have continued to evolve smoothly without the stalls and reversals described above. This also closely resembles the actual education trends in countries such as Ethiopia that did not experience cohort educational discontinuity.

**Fig. 4.**
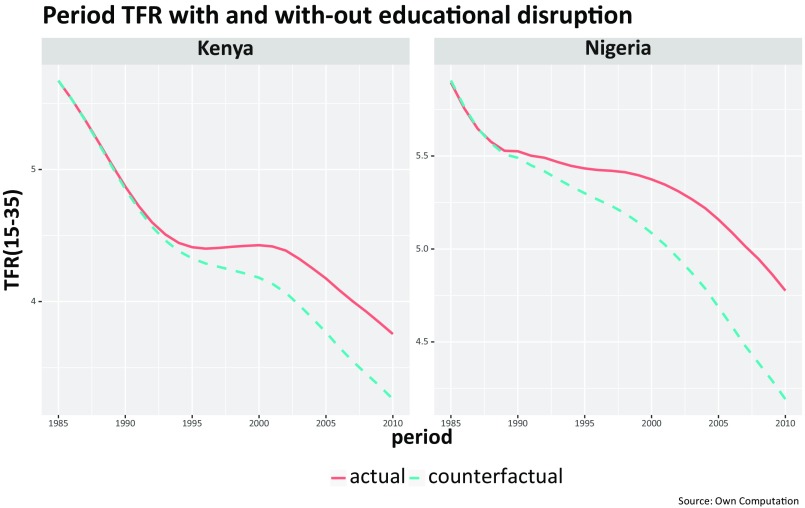
Reconstructed actual trends in TFRs (for women aged 15 to 35 y) for Kenya and Nigeria (red line) and the counterfactual trends (blue line) calculated by combining the extrapolated education trends for the cohorts born after 1970 with the observed education-specific fertility rates.

In a next step, we then applied the empirically observed age- and education-specific fertility rates (as given in Fig. 2) to the forecasted proportion of women by education groups and derived a counterfactual period total fertility rate (TFR) plotted in Fig. 4. Table 1 also lists the empirically observed and counterfactual TFRs for all African countries included in this analysis, listing the 10 countries that have been classified as fertility stalled at the top. For 2005, the biggest difference between the two rates is observed in Cote d’Ivoire, accounting for 0.50 children per woman, followed by Nigeria with 0.47. By 2010, the difference increased to 0.59 in Nigeria, followed by 0.49 in Kenya. Except for Democratic Republic of the Congo, the fertility difference induced by the education stall is larger than 0.25. As expected, for the countries not classified as having a stalled fertility decline, the difference is much lower or even negative, presumably because there also had been no discernible education stall.

**Table 1.** Reconstructed actual trends in period TFRs (for women aged 15 to 35 y) and the counterfactual trends calculated by combining the extrapolated education trends with the observed education-specific fertility rates

Country	2005			2010		
	Actual	Counterfactual	Difference	Actual	Counterfactual	Difference
Côte d’Ivoire	4.51	4.01	0.50	3.67	3.29	0.38
Cameroon	4.43	4.08	0.35	4.03	3.62	0.41
Democratic Republic of the Congo	5.51	5.31	0.20	5.21	5.03	0.18
Republic of the Congo	4.15	3.89	0.26	4.23	3.92	0.31
Kenya	4.17	3.77	0.40	3.75	3.26	0.49
Niger	7.65	7.39	0.26	6.78	6.50	0.28
Nigeria	5.16	4.69	0.47	4.78	4.19	0.59
Tanzania	5.00	4.65	0.35	4.84	4.44	0.40
Zambia	4.86	4.46	0.40	4.46	4.11	0.35
Zimbabwe	3.61	3.38	0.23	3.68	3.41	0.27
Benin	4.59	4.28	0.30	4.02	3.84	0.18
Burkina Faso	4.79	4.95	−0.17	4.13	4.43	−0.30
Ethiopia	4.93	5.02	−0.08	4.33	4.53	−0.20
Gabon	3.55	3.37	0.18	3.55	3.37	0.18
Ghana	3.62	3.63	−0.01	3.32	3.43	−0.10
Guinea	5.94	5.71	0.23	4.72	4.68	0.04
Malawi	4.86	4.94	−0.08	4.25	4.37	−0.12
Uganda	5.37	5.35	0.02	4.82	4.91	−0.09

The 10 countries listed in the top portion of the table have been classified as fertility stalled.

In sum, the difference between these counterfactual TFRs and the observed ones lies only in the weights given to the three education groups when aggregating to total fertility. Over time, these listed fertility differentials could sum up to large numbers of additional births due to the education stalls these countries had experienced in the 1980s. Translated into absolute numbers of births for all of the 10 fertility-stalled countries, between 1995 and 2010, about 13 million fewer babies would have been born to women aged 15 to 34 y. For Nigeria alone, the difference is 6.5 million births.

## Education, Cohort, and Period Effects on Fertility

In addition to the above-described simulations, we conducted multivariate analyses on the pooled individual-level dataset of all surveys for all countries and periods. The details of the different model specifications and numerical results are given in *SI Appendix*. As dependent variable for this analysis, we took the cumulative number of children that women had by age 25 y. This choice was made as a compromise between the need to relate the fertility experience studied as closely as possible to a specific time period and the need to capture, as much as possible, the quantum of fertility rather than differences in timing. The period conditions associated with this fertility indicator are the ones that prevailed when the women were 15 to 20 y old, assuming that there is some lag in the process. In terms of the age at which cumulative fertility is assessed, we also did sensitivity analysis for ages 30 and 35 y, which showed qualitatively similar results.

After experimenting with a number of different available indicators of national socioeconomic period conditions that would also cover possible cuts in reproductive health and general health care delivery systems, we chose gross domestic product (GDP) per capita as the most consistently available and most frequently used such indicator. We operationalized GDP changes over time in such a way that average growth rates over 5-y intervals were categorized as normal when they stayed within the band of ±2%, as negative when they fell below this band, and as positive when they fell above this band. In addition, the models include three levels of educational attainment assessed at the level of each individual woman by age 25 y, as well as the 5-y birth cohorts to which the women belong, to capture the above-described cohort effects. All of the results of this multivariate analysis based on different models are given in *SI Appendix*.

The results of these multivariate models over the reproductive experience of over 670,000 women in sub-Saharan Africa clearly support the above findings based on more descriptive analysis and aggregate-level simulations. Mothers’ education clearly comes out as the most significant determinant of individual-level fertility, with the estimated odds ratio indicating that women with completed primary education have, on average, 64% of the level of fertility of uneducated women, even after controlling for cohort membership and period changes in GDP.

## Conclusions

Due to the great momentum of population changes, the prospects for future population growth in Africa and consequently for the world as a whole greatly depend of the fertility trajectory in Africa over the coming years. For assessing the likely future fertility trends of Africa, it greatly matters what general approach is taken to population projections and, in particular, whether heterogeneity of the population with respect to its changing education composition is explicitly taken into account. The projections produced by the United Nations Population Division only consider the age and sex structure of the population and base their assumptions about future fertility trends on an extrapolative statistical model of the overall TFR, which is sensitive to recent trends in the TFR. The medium scenario of the most recent assessment of 2015 projects that the number of people on the planet will rise to 9.8 billion in 2050 and that sub-Saharan Africa will be responsible for more than half of the world’s population growth over the next 35 y (17). Compared with the 2010 United Nations revision, these recent projections result in world population that is 0.5 billion larger in 2050. The difference in the projection outcomes stems in large part from the extrapolation of the recent trends in fertility levels in many sub-Saharan African countries that experienced slowed or stagnating fertility declines as discussed in this paper. However, the relevance of these stalls for future fertility trends greatly depends on the nature and causes of the stalls and on the question of whether they were just temporary irregularities or more persistent.

In the above-given analysis, we have found strong empirical support for the hypothesis that this fertility stall aligned in part with a temporary stall in the education of female cohorts born in the late 1970s and 1980s. We have suggested that for most of the countries experiencing fertility stalls around 2000, there have been stalls in the education improvement of the female cohorts that entered the prime childbearing ages around that time. Detailed analyses of cohort-specific patterns and multivariate models including possible macrolevel period effects also indicate that the exceptional education experiences of the cohorts born around 1980 could indeed be associated with the observed fertility stalls. Because the more recent cohorts of young women have again picked up in terms of education, this finding suggests that in the future, we may expect an acceleration of the fertility decline as the subsequent better-educated cohorts of women move into the main childbearing ages. This finding is also relevant for the ongoing discussion as to whether population projections should be carried out by breaking down only by age and sex or whether educational attainment should be routinely included as a third demographic dimension (18, 19). The evidence discussed in this paper illustrates well that, in the case of education discontinuities, the assumed future fertility trajectories are different when heterogeneity by level of education is explicitly factored into the model compared with disregarding this heterogeneity and only observing aggregate TFR trends.

The most recent findings from the Kenya DHS are a point in case. The TFR in Kenya in the late 1970s was around 8.1 children—considered to be the highest in the world. After an initial decline, it remained stagnant for about a decade around 2000 at a level of 4.6 to 4.9 children. But most recently, the TFR experienced a rather steep dive to 3.9 children for the 3 y preceding the 2014 DHS and even 3.7 in the 2015 Malaria Indicator Survey (20). No statistical extrapolation model based on TFR alone could predict this recent decline. The education-specific analysis discussed in this paper makes it plausible because the female cohorts that experienced the stall in education expansion are being replaced in the prime childbearing ages by new and better-educated cohorts.

While the evidence for this education–fertility link at the cohort level seems to be robust, evidence for directly blaming the IMF-initiated SAPs for the education stalls seems less certain, despite the fact that many authors draw clear and strong connections between these programs and worsening health and education outcomes in the countries affected (21–26). While such direct connections are not implausible, we want to be more cautious in drawing conclusions due to the lack of reliable statistical information about how precisely the SAPs in individual countries led to cuts in the education budgets and how these cuts trickled down to effects on school enrolment rates. These austerity programs were implemented in response to economic turmoil, and it is impossible to sort out whether the SAPs or the preceding dire situations (or a combination of both) are the reasons for the evident slow-downs in educational improvement that we observe in our cohort approach. But whatever the precise reasons are, the education discontinuities described in this paper will not only be relevant in terms of their impact on fertility but also could affect the future health and income of the affected less-educated cohorts for the rest of their life courses.

Africa’s future population growth will be relevant for the rest of the world. Whether it will “only” increase to two billion as shown by optimistic scenarios that assume successful implementation of the sustainable development goals (27, 28), or by more-pessimistic scenarios assuming slow or stalled development and thus resulting in four to five billion Africans by the end of the century, Africa’s future population growth will first of all impact on the future of living conditions and quality of life of Africans. But it will also affect other continents due to out-migration pressure, global environmental impacts, and, of course, the efforts needed to live up to the promise to eradicate poverty, hunger, and premature death in all corners of the planet. Continued rapid population growth will make this an up-hill battle. Education is currently underfunded, particularly in Africa (29, 30). A new effort for massive investment in education, particularly of girls, will not only help to slow this growth but will also empower Africans and create the human capital needed for rapid social and economic development and sustainable increases in human well-being.

## Supplementary Material

Supplementary File

Supplementary File
