# Supplementary material for: Stalls in Africa’s fertility decline partly result from disruptions in female education
Source: Proc Natl Acad Sci U S A. 2019 Feb 4;116(8):2891–6. doi: 10.1073/pnas.1717288116 (PMC6386713; doi:10.1073/pnas.1717288116)
Supplement: Supplementary File [file pnas.1717288116.sapp.pdf]

PNAS
www.pnas.org

Supplementary Information for

Stalls in Africa's fertility decline party result from disruptions in female education

Endale Kebede, Anne Goujon, Wolfgang Lutz

Wolfgang Lutz
Email: lutz@iiasa.ac.at

This PDF file includes:

Supplementary text
Figs. S1 to S5
Tables S1 to S3

www.pnas.org/cgi/doi/10.1073/pnas.1717288116

Table S1: Variable definitions and sources

Variable /Indicator	Definition	Source
Growth of GDP/capita	Annual percentage change in gross domestic product (GDP) per capita. Computed by the authors from the available annual GDP/capita values (1960-2016)	Word development indicators (WDI) 2017
Cohort ASFR	Age specific fertility rates (ASFR) by single year of age and 5-year birth cohort of women	Computed from Demographic and health surveys (DHS)
Cohort education	Share of women by cohort with no formal education, primary education or at least completed primary education	DHS
$Cohort_{c,i}$	Birth cohort of women i of country c (<1960, 1960-64,1965-69,1970-74,1975-79,1980-84)	DHS

I. Reconstructing Fertility Trends from Pooled Birth Histories

The retrospectively collected Demographic and health surveys were used to reconstruct fertility trends by age and educational attainment of the mother. Since DHSs are sample surveys, the information gathered at different points in time for the same cohorts of women is not necessarily identical. Appendix Figure S.1 shows the cohort fertility rate for Kenya from different surveys. It shows that, particularly for earlier cohorts, one could get different figures of cohort fertility rates for the same cohort interviewed at different points in time. Thus, reconstructing fertility trends by simply pooling birth histories from different surveys would be misleading.

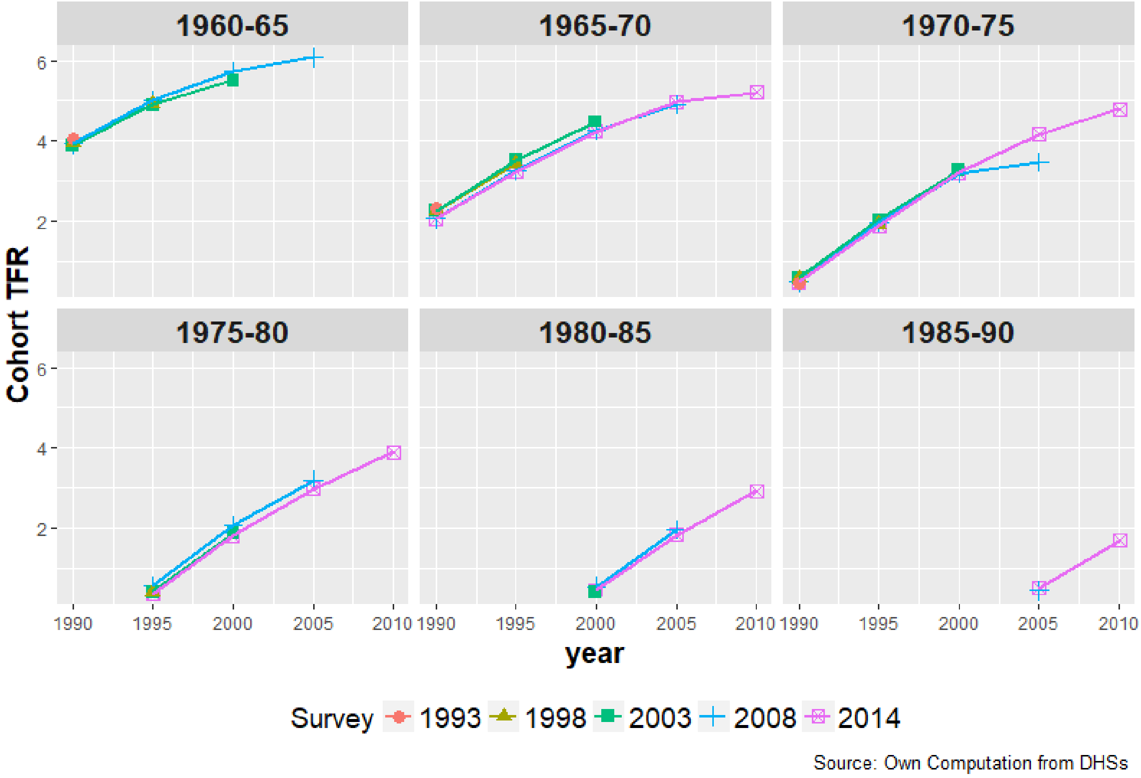

Figure S1: Cumulative total fertility rates by survey-year and five-year birth cohorts of women in Kenya

The mismatch is even stronger for women without any education (Figure S2). This can be due to the fact that the so called ‘recall-bias’, is negatively associated with the educational attainment of women.

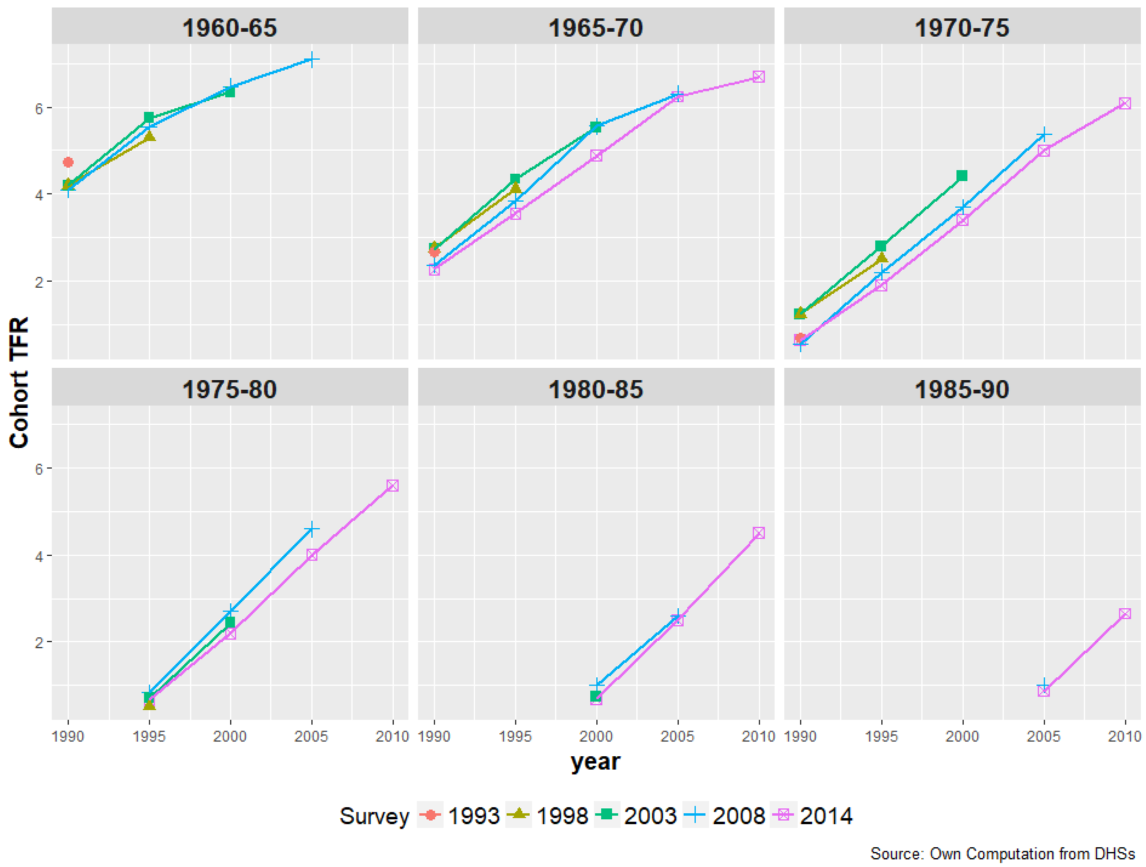

Figure S2: Cumulative total fertility rates by survey-year and five-year birth cohorts of women with no education in Kenya

Hence, in the case that different surveys provide different information on fertility for the same cohorts a weighted average of the different surveys was used. First, for each survey, birth events and exposures were computed by single year of age, birth year and education category of women. Second, the weighted average of events and exposures of different surveys were computed for each age, birth cohort and education category of women. Third, the age and education specific averaged events and exposures were smoothed using spline functions across ages and birth cohorts. Finally, the age and education specific cohort fertility rates were computed from the smoothed weighted average of events and exposures. The result is presented in Figure S3.

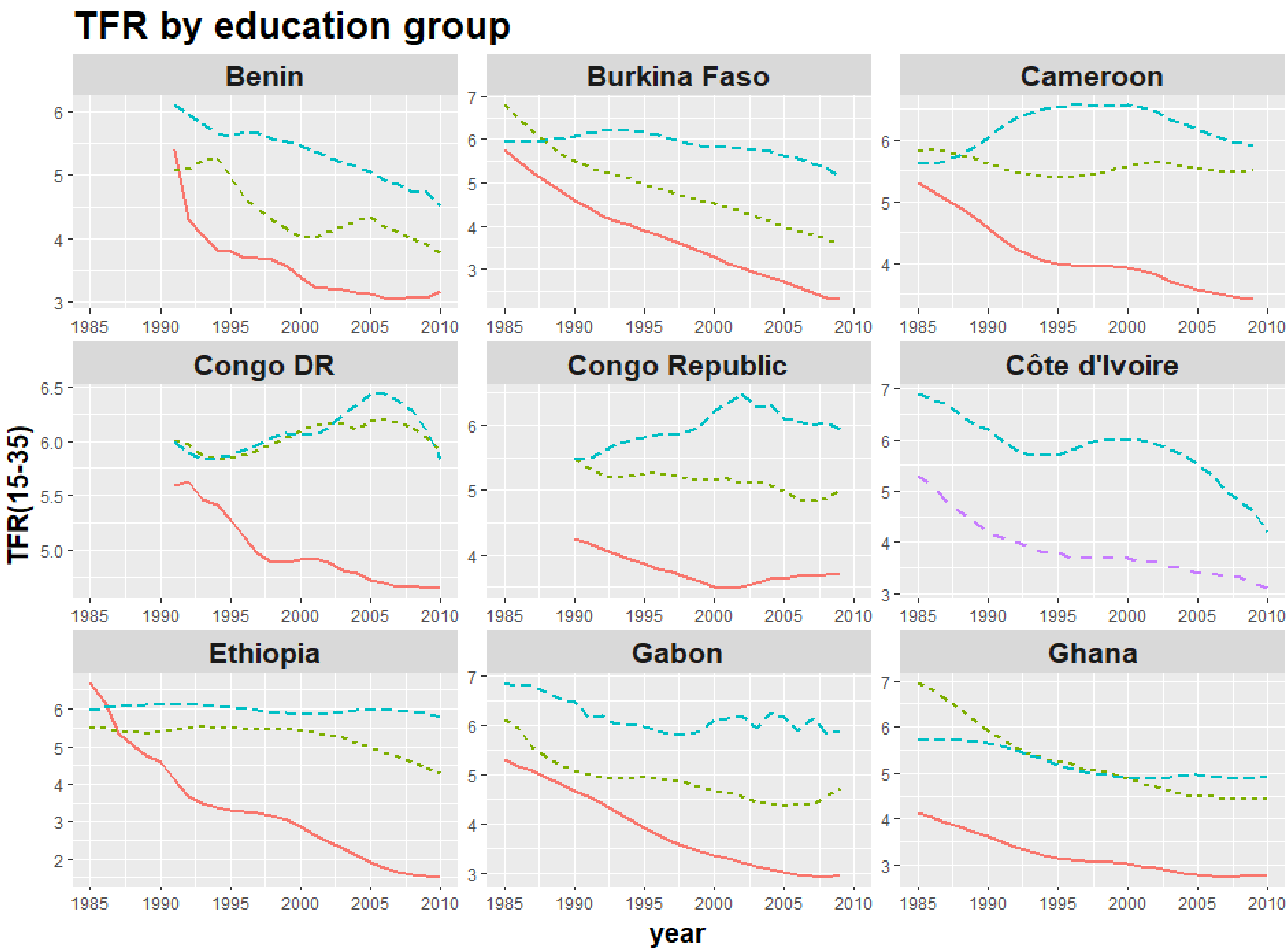

Source: Own Computation

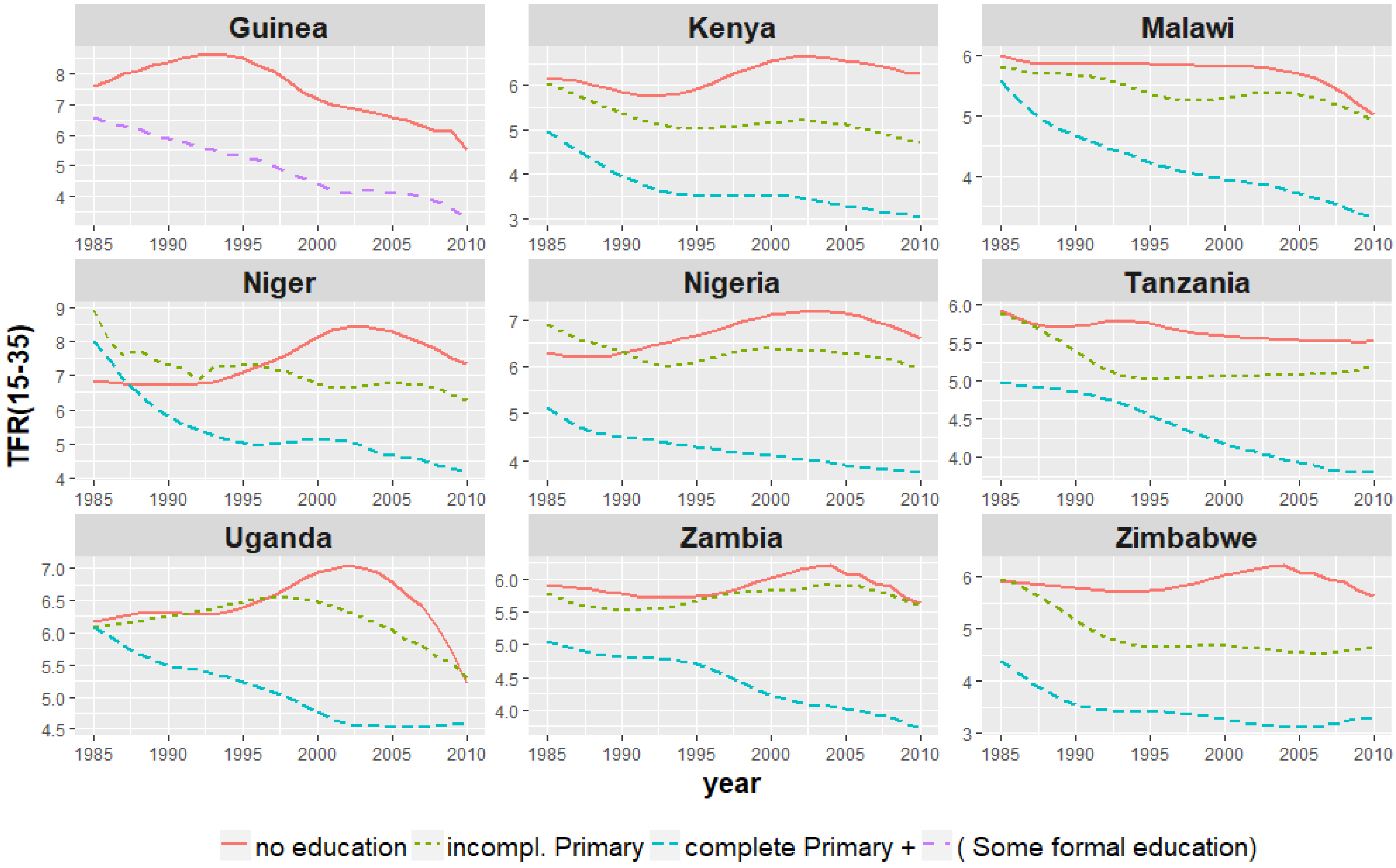

Figure S3: Period total fertility rates (1985-2010) by education category (no education, incomplete primary, completed primary or more. For Côte d'Ivoire and Guinea, we have too few cases for women with completed primary education or more, and the counterfactual analysis is based on trends of two education groups only: no education and some formal education.

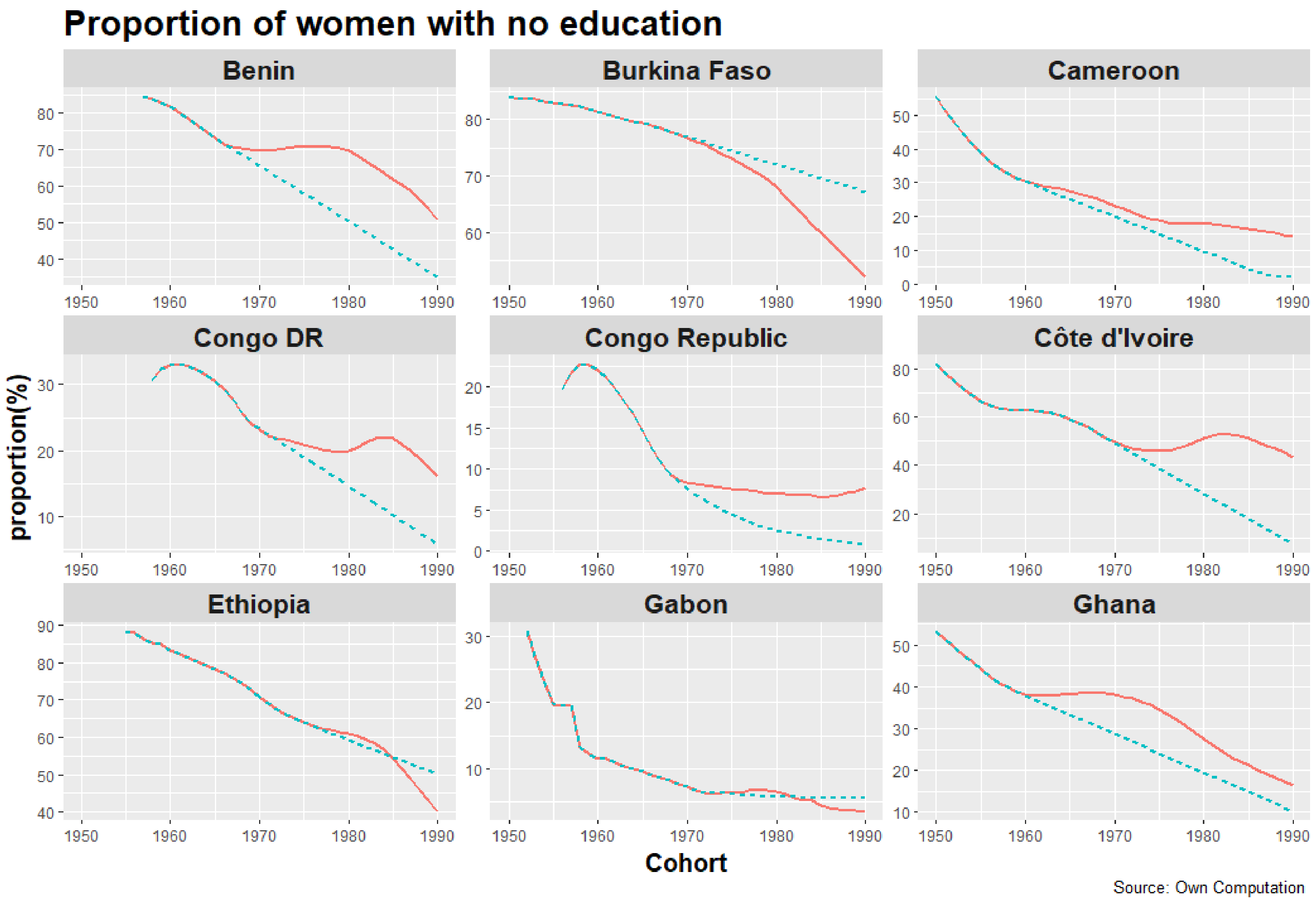

Proportion of women with no education
Benin
Burkina Faso
Cameroon
Congo DR
Congo Republic
Côte d'Ivoire
Ethiopia
Gabon
Ghana
proportion(%)
Cohort
Source: Own Computation

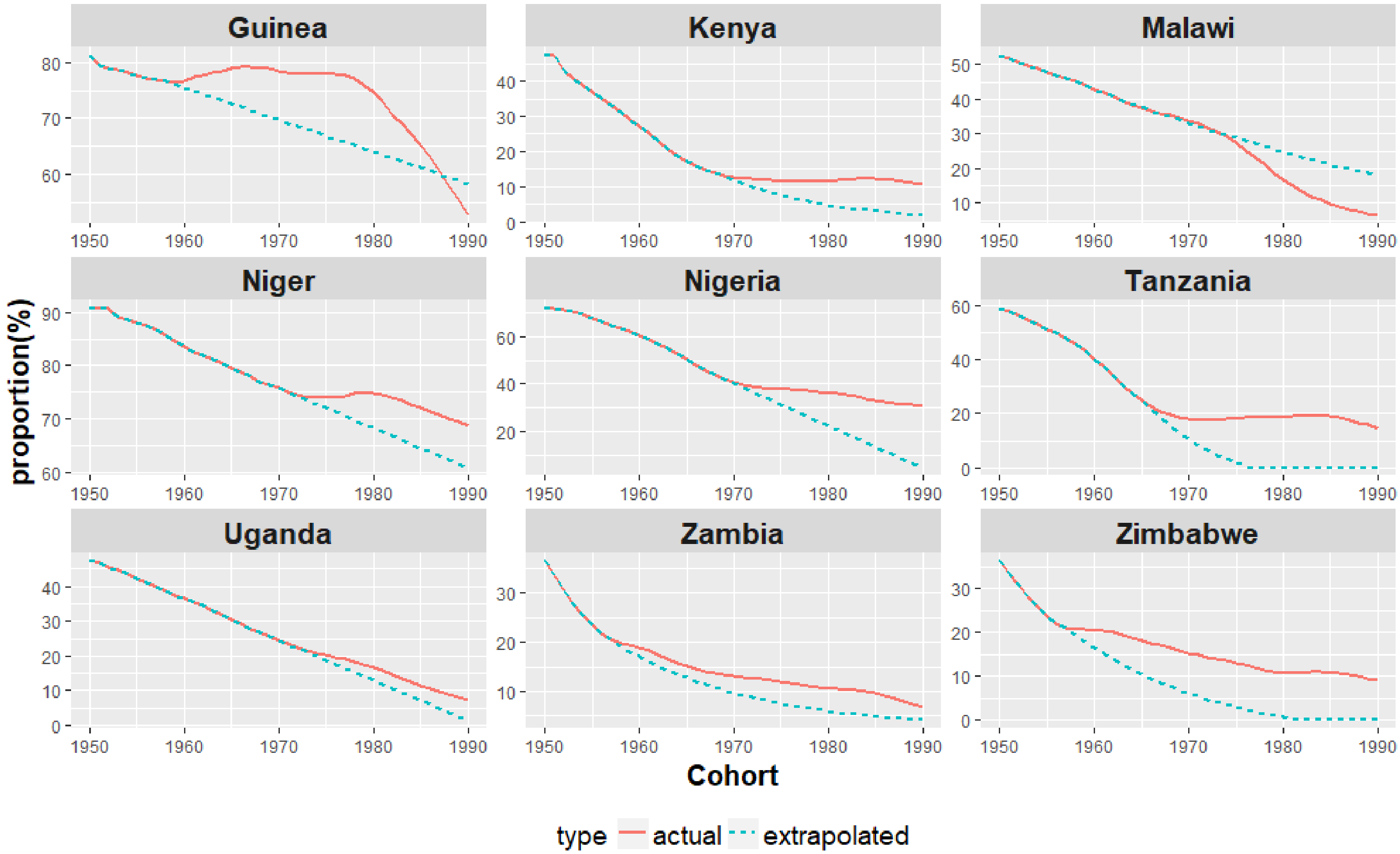

Figure S4.1: Reconstructed proportions of women with no formal education by cohorts born between 1950 and 1990 (red line) and extrapolated trends based on a hypothetical continuation of the trend of the earlier period (blue line).

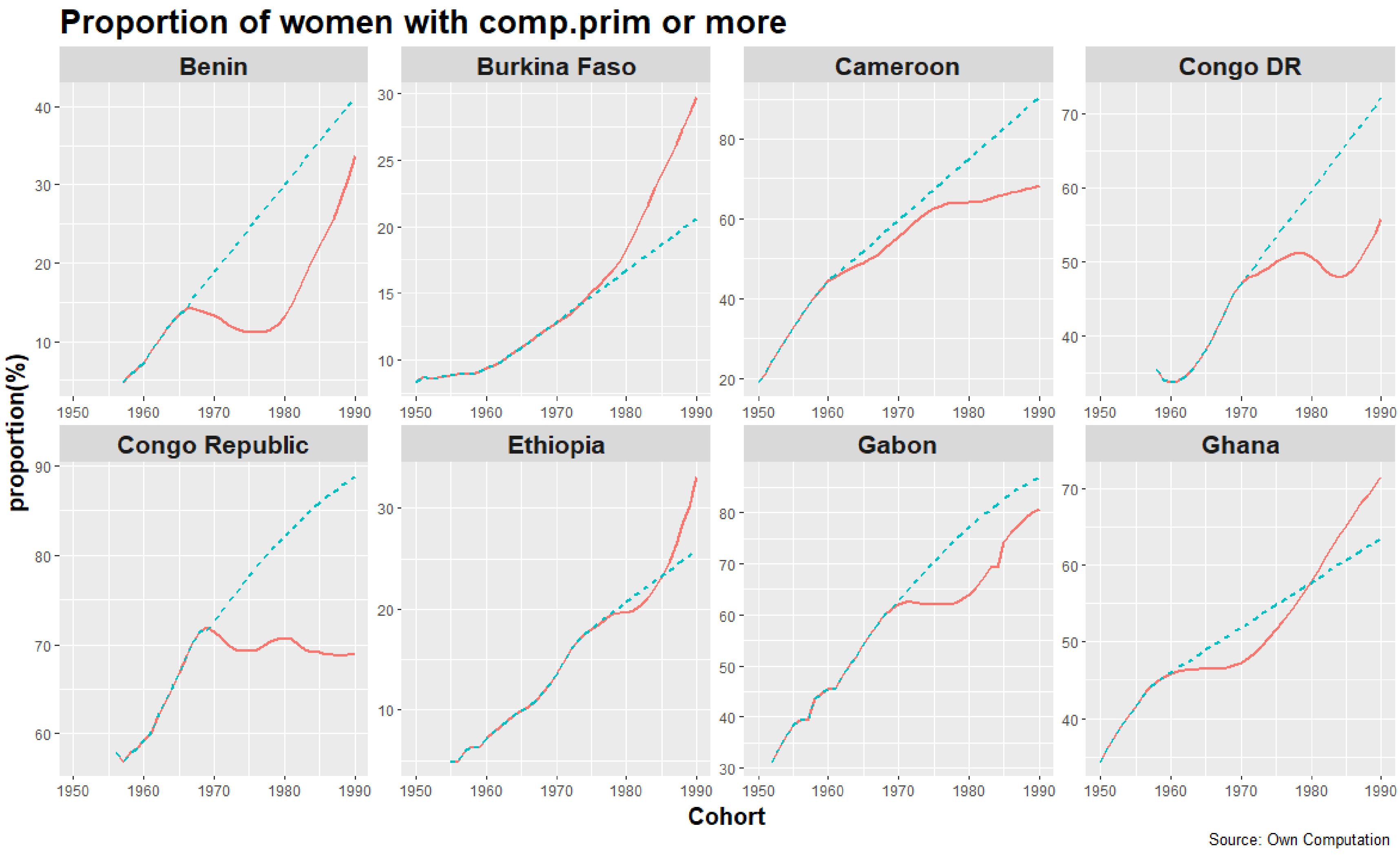
Proportion of women with comp.prim or more
Benin
Burkina Faso
Cameroon
Congo DR
Congo Republic
Ethiopia
Gabon
Ghana
proportion(%)
Cohort
Source: Own Computation

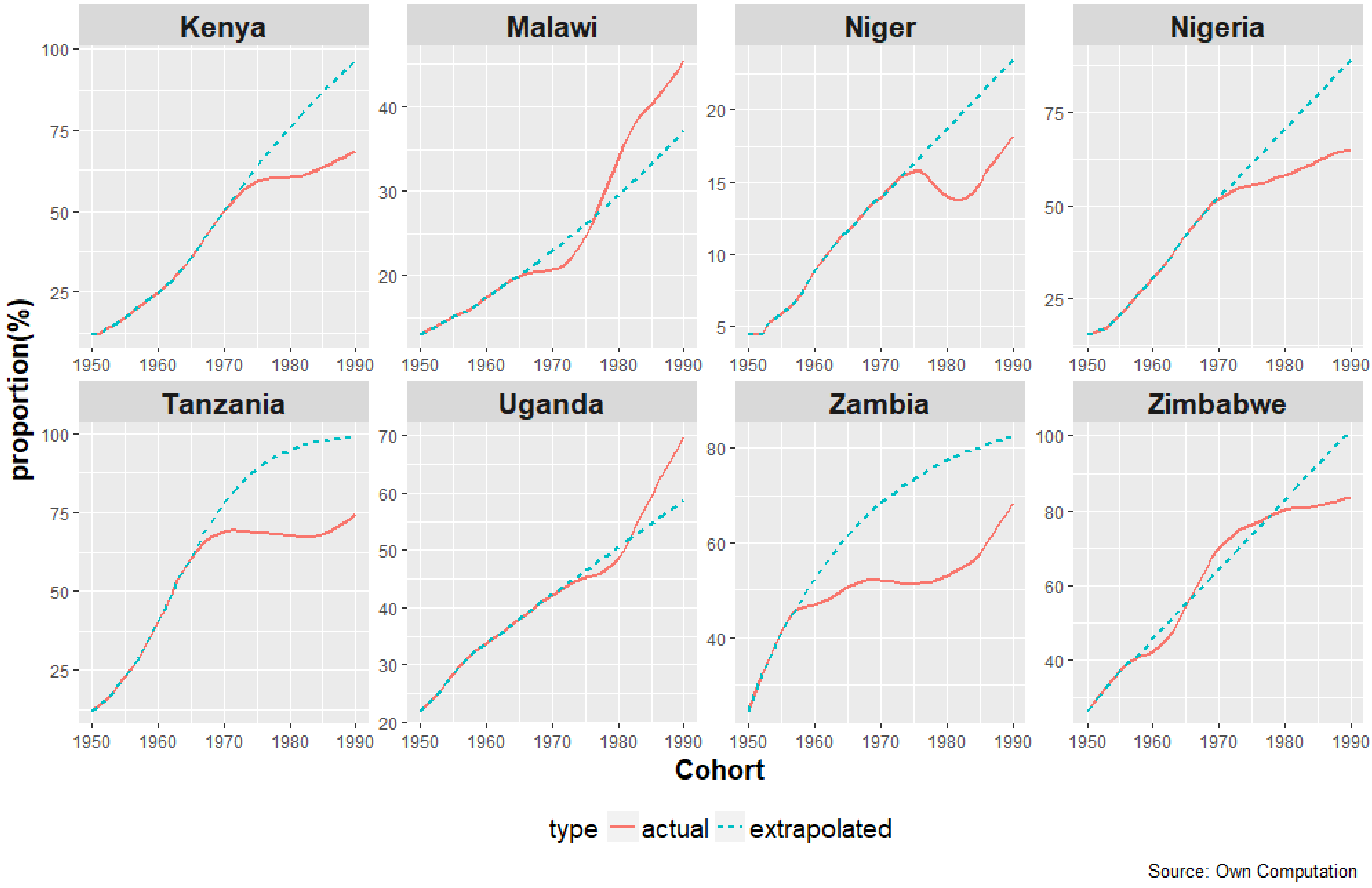

Figure S4.2: Reconstructed proportions of women with completed primary education or more education, by cohorts born between 1950 and 1990 (red line) and extrapolated trends based on a hypothetical continuation of the trend of the earlier period (blue line).

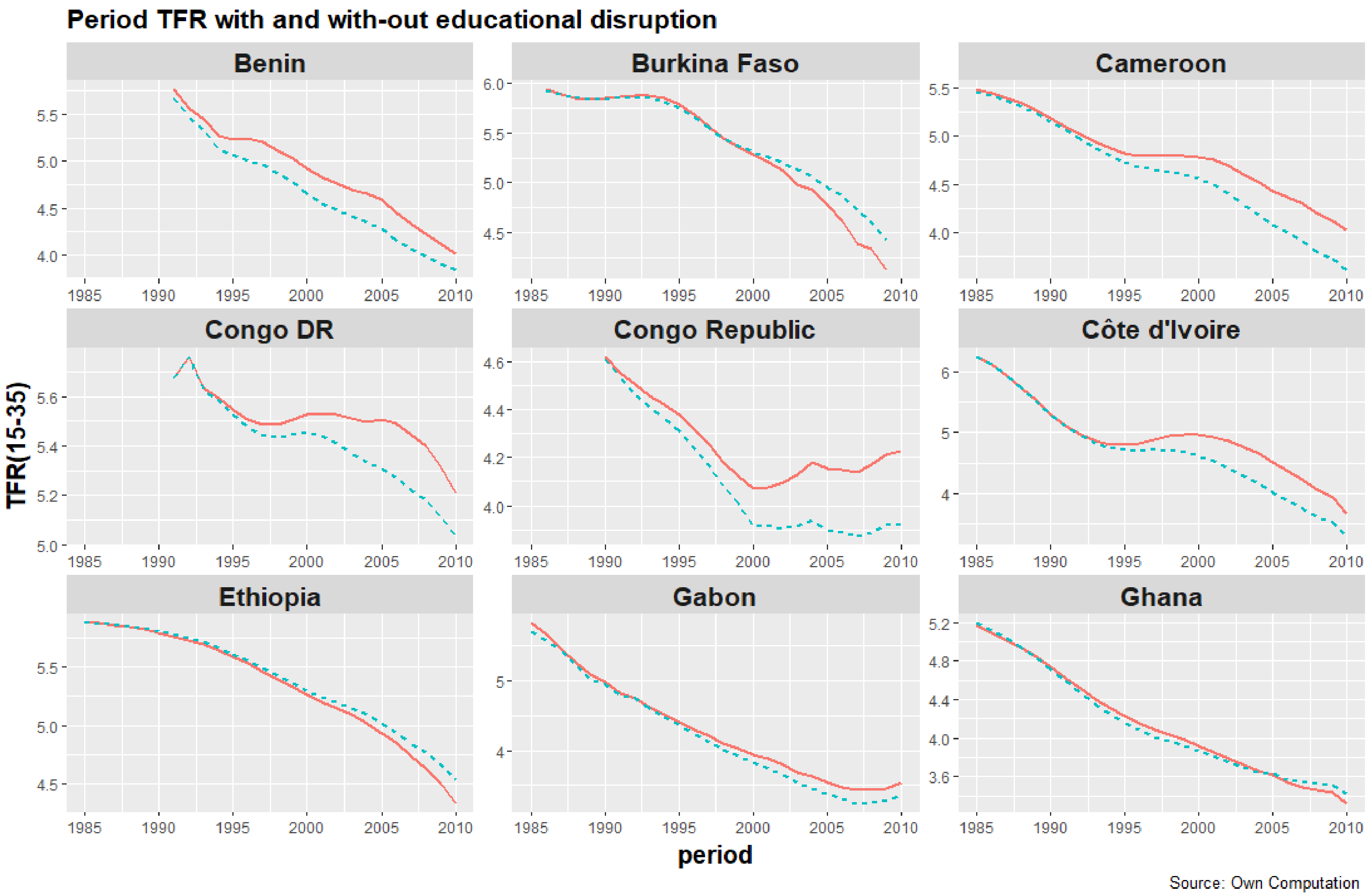
Period TFR with and with-out educational disruption
Benin
Burkina Faso
Cameroon
Congo DR
Congo Republic
Côte d'Ivoire
Ethiopia
Gabon
Ghana
TFR(15-35)
period
1985
1990
1995
2000
2005
2010
Source: Own Computation

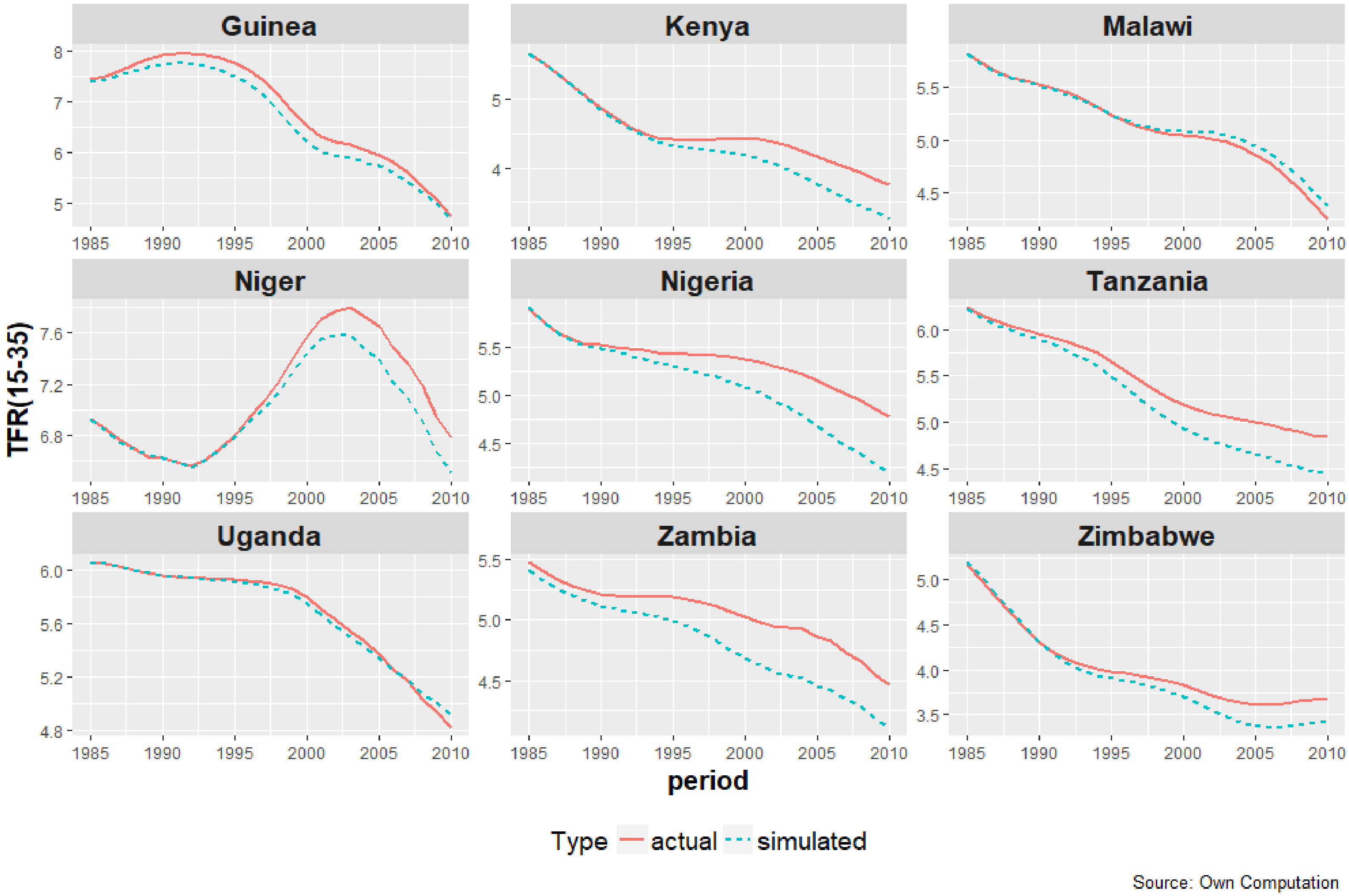

Figure S5. Reconstructed actual trends in period total fertility rates (for women aged 15-35) (red) and the counterfactual trends (blue) calculated by combining the extrapolated education trends with the observed education-specific fertility rates.

II. Model Specifications

The Mixed Effect Poisson Model

In order to shed light on the relative importance of cohort and period effects on fertility changes, we assess their respective influence on early childbearing by estimating a model explaining the cumulative number of births to women up to the age of 25.

Our base model is specified in the following way:

$$\log\left(\mathrm{CNB25}_{i,c,t}\right) = \alpha + \beta 1 Cohort_{c,i} + \beta 2 Educ_{c,i} + \beta 3 Growth\ GDP_{c,t-1} + U_c$$

It is a mixed effect Poisson regression model with woman i nested in country c. The dependent variable is the cumulative number of births for women i residing in country c by the time t she turns 25. The key explanatory variable $Cohort_{c,i}$ represents the five-year cohort membership of woman i residing in country c. Women's educational attainment $Educ_{c,i}$ is defined as categorical variable: no formal education, incomplete primary education, completed primary education or more. $Growth\ GDP_{c,t-1}$ represents the period economic conditions when the woman was in primary childbearing age (15-19). It is computed as the five-year moving average value (years when the woman i was age 15-19) of the growth of GDP/capita of the respective country c. It is entered into the model as a categorical variable: normal period [-2.0%, 2.0%], negative growth (-2.0% or less) and positive growth (2.0% or more). The parameter U_c corresponds to a country random effect, which assumes a normal distribution with constant variance.

Three related duration models have been estimated and compared. First, in the 'Bi-variate model', we have constructed a mixed-effects model including the three predictors (cohort membership, women's education and GDP growth) as fixed effects, separately. This allows understanding the pattern of inter-cohort progress in fertility without controlling for education and period related factors. Second, in 'Model-II', we added the partial effect of education by including an education variable as an additional individual-level predictor. Finally, the net effect and robustness of education and growth of GDP/capita are compared in the 'Model-III' that includes individual cohort membership, individual education, period-related macro-factor (growth of GDP/capita) as fixed effects. The full-estimated models are presented in the appendix tables S.2-S.3.

Mothers' education is revealed as the most significant determinant of individual level fertility with the estimated odds ratio indicating that women with completed primary education have on average 64 percent of the level of fertility of uneducated women even after controlling for cohort membership and period changes in GDP.

The effect of membership in birth cohorts without controlling for other factors indicates the expected decline over time even for the cohorts born after 1975 when all 18 countries are considered together (table S.2). When only the countries with stalled fertility are considered (table S.3) the cohort effects also show no further decline and even a mild increase for the cohort born in 1980-85. Once education is also included in the model, the cohort effect after 1970 disappears completely. This is true for all countries studied together and the stalled countries studied separately. It indicates that education indeed explains most of the observed inter-cohort differences in fertility.

Changes in GDP/capita taken as indicator for changing national level period conditions show a rather complex pattern. When looking at the bi-variate association, both periods of higher than usual and lower than usual (outside the 2 percent band) seem to be associated with moderately higher fertility. But when education and cohort membership are accounted for the period effect of GDP changes on fertility completely disappears. This statistical insignificance of GDP, however, should not be taken as proof for the absence of period effects in fertility stall. The increases in fertility levels among the least educated groups of women in the affected countries clearly suggest that something was changing there. In the context of our study this finding just indicates that GDP – although being the most widely and consistently available indicator of changing macro-level conditions – is probably not a good proxy for period economic. Unfortunately, all attempts to find better indicators such as measures of national level changes in expenditure on family planning suffered from the lack of consistent empirical information. Hence, we have to suffice with the more speculative qualitative statement that some period changes could have resulted in higher fertility of the least educated and most vulnerable groups of women, an assessment that is in line with a recent comprehensive study of African fertility trends (17).

Variables	Bi-variate		Model-II		Model-III	
	**IRR**	**95% CI**	**IRR**	**95% CI**	**IRR**	**95% CI**
**Birth cohort**						
<1960	1.137	1.129-1.146	1.050	1.042-1.058	1.050	1.042-1.058
1960-64	1.103	1.095-1.111	1.061	1.053-1.070	1.061	1.053-1.070
1965-69	1.051	1.044-1.059	1.038	1.031-1.045	1.038	1.031-1.045
1970-74(Reference)	1.00		1.00		1.00	
1975-80	0.983	0.976-0.990	0.995	0.988-1.002	0.995	0.988-1.002
1980-84	0.978	0.971-0.985	0.999	0.991-1.006	0.998	0.991-1.006
**Women's education**						
No formal education(ref)	1.00		1.00		1.00	
Some primary	0.931	0.926-0.936	0.936	0.931-0.941	0.936	0.931-0.941
Completed primary+	0.637	0.634-0.641	0.645	0.641-0.649	0.645	0.641-0.649
**Growth of GDP/capita (%)**						
[-2.0,2.0] (reference)	1.00				1.00	
Lower than -2.0	1.014	1.008-1.021			0.998	0.992-1.005
Above 2.0	1.034	1.028-1.039			1.000	0.994-1.006
**Random effects**	**MOR**		**MOR**		**MOR**	
Level 2(Country)	**1.11**		1.11		1.11	
No. of country	**18**		18		18	
No. of women	**439,132**		**439,132**		**439,132**	

Table S2. Mixed effect Poisson regression results: Estimated incidence rate ratio (and 95% confidence intervals) for the number of children ever born by age 25 for women aged 25 or more at the time of the survey. IRR below 1 shows the smaller number of cumulative number of births relative to the reference group.

Variables	Bi-variate		Model-II		Model-III	
	**IRR**	**95% CI**	**IRR**	**95% CI**	**IRR**	**95% CI**
**Birth cohort**						
<1960	1.171	1.161-1.181	1.085	1.076-1.094	1.075	1.065-1.084
1960-64	1.114	1.105-1.124	1.069	1.060-1.079	1.061	1.051-1.070
1965-69	1.042	1.034-1.051	1.030	1.021-1.038	1.025	1.016-1.034
1970-74(Reference)	1.000		1.00		1.00	
1975-80	0.988	0.980-0.996	0.993	0.985-1.002	0.995	0.987-1.004
1980-84	0.986	0.977-0.996	0.992	0.983-1.002	0.996	0.986-1.003
**Women's education**						
No formal education(ref)	1.000		1.000		1.000	
Some primary	0.967	0.960-0.974	0.973	0.966-0.980	0.973	0.966-0.980
Completed primary+	0.730	0.726-0.735	0.744	0.739-0.749	0.744	0.739-0.749
**Growth of GDP/capita (%)**						
[-2.0,2.0] (reference)	1.000				1.000	
Lower than -2.0	1.026	1.019-1.033			1.016	1.009-1.024
Above 2.0	1.121	1.113-1.129			1.029	1.021-1.038
**Random effects**	**MOR**		**MOR**		**MOR**	
Level 2(Country)	**1.10**		1.10		1.10	
No. country	**10**		10		10	
No. of women	**252,143**		**252,143**		**252,143**	

Table S3. Mixed effect Poisson regression results: Estimated incidence rate ratio (and 95% confidence intervals) for the number of children ever born by age 25 for women aged 25 or more at the time of the survey. Only 'fertility stalled' countries are included. IRR below 1 shows the smaller number of cumulative number of births relative to the reference group.
